# Biphasic regulation of autophagy by miR-96 in prostate cancer cells under hypoxia

**DOI:** 10.18632/oncotarget.2396

**Published:** 2014-08-27

**Authors:** Yi Ma, Hao-Zheng Yang, Bai-Jun Dong, Han-Bing Zou, Yan Zhou, Xian-Ming Kong, Yi-Ran Huang

**Affiliations:** ^1^ Department of urology, Renji Hospital, School of Medicine, Shanghai JiaoTong University, Shanghai, China; ^2^ Department of biobank, Renji Hospital, School of Medicine, Shanghai JiaoTong University, Shanghai, China; ^3^ Department of central laboratory, Renji Hospital, School of Medicine, Shanghai JiaoTong University, Shanghai, China

**Keywords:** Autophagy, Hypoxia, microRNAs, Oncogene, Prostate Cancer

## Abstract

Autophagy favors cell survival under hypoxia, and increasing evidence revealed that microRNAs regulate autophagy. We report here hypoxia increased the expression of miR-96 in prostate cancer cells, and miR-96 stimulated autophagy by suppressing *MTOR*. We found that inhibition of miR-96 abolished hypoxia-induced autophagy. Paradoxically, ectopic over-expression of miR-96 to a certain threshold, also abolished the hypoxia-induced autophagy. Further studies have shown that high levels of miR-96 inhibited autophagy through suppressing *ATG7*, a key autophagy-associated gene. Importantly, the miR-96 expression level threshold was determined, and the effects of miR-96 on autophagy on either side of the threshold were opposite. These data demonstrate hypoxia-induced autophagy is at least partially regulated by miR-96; miR-96 can promote or inhibit autophagy by principally inhibiting *MTOR* or *ATG7* depending on the expression levels of miR-96. Our observation might reveal a novel regulatory mode of autophagy by microRNAs under hypoxia.

## INTRODUCTION

Prostate cancer is the most common cancer in Western men and its rate is increasing in the Eastern world [1]. During the multiple-step process of tumorigenesis, a typical prostate cancer cell has accumulated around 100 genetic and epigenetic alterations of its genome [2, 3]. It is now widely believed that tumor microenvironment plays key role in the carcinogenesis and resistance of cancer cells to anti-cancer drugs. Due to the transient and long-term lack of nutrients, energy and oxygen, cancer cells frequently undergo hypoxia and nutrient deprivation. It has been well documented that hypoxia enhances tumorigenesis by promoting genome instability [4, 5]. Moreover, hypoxia is currently becoming an important prognostic marker in prostate cancers [6], which indicates that hypoxia may cause molecular changes that contribute to the proliferation or differentiation of prostate cancer cells. However, the mechanism by which hypoxia affects the survival of prostate cancer cells remains to be determined.

It was recently discovered that hypoxia induces autophagy, providing a mechanism of the protection of prostate cancer cells by hypoxia [7]. Autophagy is a tightly controlled process that eliminates defective organelles and proteins from the cell. Classic macroautophagy initiates from an isolation membrane (phagophore), followed by the formation of a double-membrane autophagosome, which in turn fuses with lysosomes. Autophagy is mainly controlled by *MTOR* signaling and *ATG* family members. Recently, autophagy has been found as a key regulator of cancer cell survival. By providing nutrition and reducing oxidative stress, autophagy contributes to sustained growth of various types of tumor cells [8-10]. In contrast, deregulation of autophagy results in metabolic imbalance and cell death [11]. Autophagy mediates lipid droplet degradation and lipolysis, which promotes the survival of prostate cancer cells [12]. Furthermore, the combinatory treatment of autophagy inhibitors and anticancer drugs has a more significant inhibitory effect on prostate cancer growth [13, 14]. However, it is still unknown how autophagy is regulated in prostate cancer under hypoxia.

It has been reported that hypoxia regulates microRNAs (miRNAs) expression [15]. miRNAs are small, noncoding RNA molecules that modulate gene expression and regulate many cellular processes [16]. miRNAs can function as tumor suppressors, oncogenes, or both. Deregulation of miRNAs has been found in most cancers. It has been demonstrated that miRNAs modulate autophagic signaling networks in cancer cells [17, 18]. These facts led us to propose that miRNAs may affect the growth and survival of cancer cells through modulating autophagy under hypoxia.

In this study, we have investigated the function of miR-96 in the regulation of autophagy in prostate cancer cells under hypoxia, and found that miR-96 regulates autophagy under hypoxia via targeting *MTOR* and *ATG7*.

## RESULTS

### Either up-regulation or down-regulation of miR-96 suppresses prostate cancer cell proliferation *in vitro* and tumor growth *in vivo* under hypoxia

**Figure 1:**
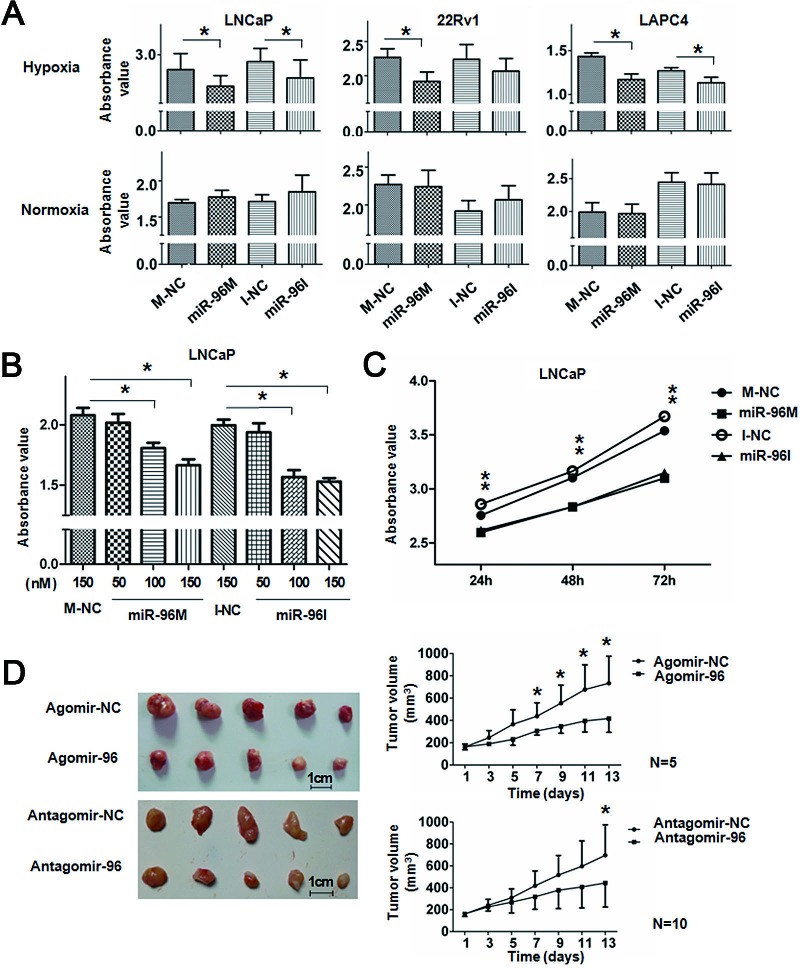
Up-regulation or down-regulation of miR-96 inhibited prostate cancer cell proliferation *in vitro* and tumor growth *in vivo* A, LNCaP, 22Rv1, and LAPC4 cells were transfected with 100nM miR-96M or miR-96I in the presence or absence of hypoxia. Cell viability was assessed by the CCK-8 assay after 36 h. 450nm absorbance value was tested. (n=6) B, LNCaP cells were transfected with miR-96M or miR-96I with the concentrations as indicated. Cell viability was determined by CCK-8 after incubation in hypoxia for 36 h. (n=6) C, LNCaP cells were transfected with 100nM miR-96M or miR-96I and incubated for the time as indicated. Cell viability was determined by CCK-8. (n=6) D, LNCaP cells were injected into mice subcutaneously. After the tumor was established, tumors were directly injected with 4nmol agomiR-96 or antagomiR-96 in 50ul PBS followed by monitoring of tumor size for 2 weeks (mean±SD), and representative tumor samples were taken. M-NC: miRNAs mimics negative control; I-NC: miRNAs inhibitors negative control. *p < 0.05

miR-96 is located at chromosome 7q32, a region containing several oncogenes including *MET* and *BRAF* and frequently amplified in cancers [19, 20]. miR-96 is up-regulated and demonstrates oncogenic activities in many common cancers, including liver [21, 22], prostate [23, 24], bladder [25] and colon cancers [26]. However, ectopic expression of miR-96 inhibited the growth of several cancer cells [27, 28], indicating a complex function of miR-96 in the initiation, progression and maintenance of tumorigenesis. In order to understand the biology of miR-96 in prostate cancer, we assayed the cell viability of prostate cancer cells in hypoxia by either up-regulating or down-regulating miR-96. Prostate cancer LNCaP, 22Rv1 and LAPC4 cells were transfected with 100nM miR-96 mimics (miR-96M) or miR-96 inhibitors (miR-96I), in the presence or absence of hypoxia. Cell viability was assessed by the CCK-8 assay after 36 h. The results showed that miR-96M significantly inhibited the cell proliferation of LNCaP, 22Rv1 and LAPC4 cells in hypoxia but not normoxia (Fig. 1A). Unexpectedly, miR-96I also significantly suppressed the proliferation of LNCaP and LAPC4 cells and slightly of 22Rv1 cells in hypoxia but not normoxia. Increase in the concentration of miR-96M or miR-96I resulted in further inhibition of LNCaP cell proliferation (Fig. 1B); however, different doses of mimics negative controls (M-NC) or inhibitors negative controls (I-NC) caused similar changes in cell survival (Fig. S1A). We next determined the viability of LNCaP cells for 24 h, 48 h and 72 h and found that enhanced inhibitory effects were observed for miR-96M or miR-96I after both 48 and 72 h in comparison to M-NC or I-NC (Fig. 1C). These results indicate that either miR-96M or miR-96I reduces the cell proliferation of prostate cancer cells in a time and dosage dependent manner under hypoxia.

To extend our observations from cell cultures, we established prostate cancer LNCaP mouse xenograft model. Intratumoral injections of agomiR-96 or antagomiR-96I significantly reduced the volumes of subcutaneous tumors (Fig. 1D), demonstrating that both agomiR-96M and miR-96I can inhibit tumor growth.

### Up-regulation and down-regulation of miR-96 abolishes hypoxia-induced autophagy

**Figure 2:**
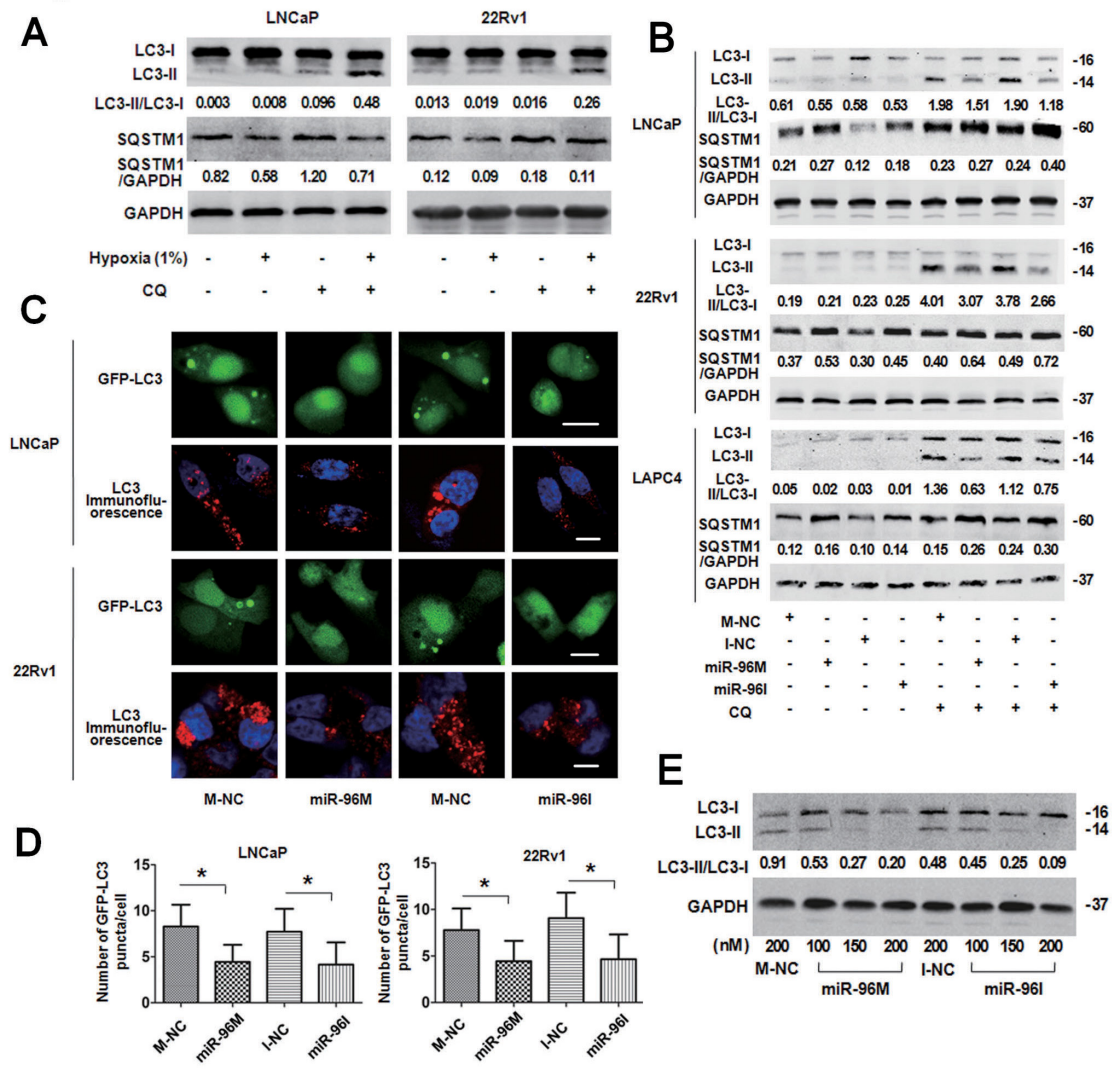
Transfection of miR-96M or miR-96I inhibited hypoxia-induced autophagy in prostate cancer cell A, LNCaP and 22Rv1 cells were exposed to normoxia or hypoxia (1%) with or without CQ (50μM and 30μM, respectively) for 24 h. LC3B, SQSTM1 and GAPDH were determined by Western blot. B, LNCaP, 22Rv1, and LAPC4 cells were transfected with 100nM miR-96M or miR-96I with or without CQ (50μM, 30μM and 30μM, respectively). After 36 h exposure to hypoxia, LC3B, SQSTM1 and GAPDH were determined by western blot. C, LNCaP and 22Rv1 cells were co-transfected with GFP-LC3 and miR-96M or miR-96I and subjected to hypoxia for 36 h. LC3 was stained for immunocytochemistry. The cells were then observed under confocal microscopy. (Scale bars: LNCaP 20 μm; 22Rv1 10 μm.) D, Number of GFP-LC3 puncta/cell was calculated with high-content screening. (n=3) E, LNCaP cells were transfected with miR-96M or miR-96I at the indicated concentrations and incubated under hypoxia for 36 h. LC3B, and GAPDH were determined by Western blot. 50 μM CQ was used in this experiment. M-NC: miRNAs mimics negative control; I-NC: miRNAs inhibitors negative control. *p < 0.05

One of the physiological responses of hypoxia is the induction of autophagy [29]. To investigate if hypoxia induces autophagy in prostate cancer cells, we detected LC3B and SQSTM1 expression level in LNCaP and 22Rv1 cells treated with hypoxia in the presence or absence of CQ (Fig. 2A). Consistent with the rapid turnover of LC3-II in prostate cancer cells [30, 31], only basal levels of LC3-II were detected in the cells in the absence of CQ. However, in the presence of CQ, cells treated with hypoxia showed increased level of LC3-II and decreased SQSTM1, which indicates hypoxia induces autophagy in these cells.

To test if miR-96 modulates autophagy under hypoxia, we treated LNCaP, 22Rv1 and LAPC4 cells with miR-96M or miR-96I with or without CQ in hypoxia for 36 h and assessed the lipidation of endogenous LC3 (Fig. 2B). Only basal levels of LC3-II were detected in the cells without treatment with CQ. However, in the presence of CQ, cells transfected with either miR-96M or miR-96I demonstrated decrease of LC3-II and accumulation of SQSTM1. To support this observation, we detected the punctate GFP-LC3 in LNCaP and 22Rv1 cells by confocal microscopy (Fig. 2C) and high-content screening system (Fig. 2D). The number of punctate GFP-LC3 per cell significantly decreased in cells transfected with either miR-96M or miR-96I (Fig. 2D). In addition, increasing the concentrations of either miR-96M or miR-96I led to further down-regulation of LC3-II (Fig. 2E), however different doses of M-NC or I-NC caused similar changes in autophagy level (Fig. S1B).

### Dose-dependent regulation of miR-96 on hypoxia induced autophagy and cell viability

**Figure 3:**
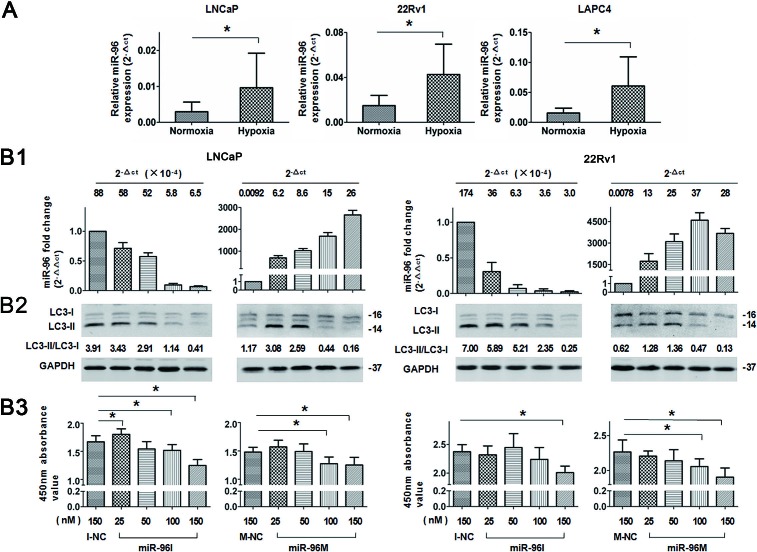
Dose-dependent regulation of miR-96 on hypoxia induced autophagy in prostate cancer cells A, LNCaP, 22Rv1, and LAPC4 cells were cultured in hypoxia for 36 h. miR-96 expression levels were determined by qPCR (n=3). B, Cells were transfected with different concentrations of miR-96M or miR-96I (25-150nM) and then exposed to hypoxia for 36 h. miR-96M expression level (B1), LC3B (B2), and cell viability (B3) were assayed by qPCR (n=3), immunoblots and CCK-8 (n=6), respectively. CQ was used in immunoblot experiments. M-NC: miRNAs mimics negative control; I-NC: miRNAs inhibitors negative control. *p < 0.05.

To further investigate the role of miR-96 in prostate cancer cells subjected to hypoxia, we first detected the relative expression level of miR-96 by qPCR in cells incubated under normoxia or hypoxia. miR-96 levels in the cells exposed to hypoxia were increased by 2-3 folds (2.1, 2.5 and 3.5 folds in LNCaP, 22Rv1 and LAPC4 cells, respectively, *p*<0.05) as compared with the normoxic groups (Fig. 3A). In order to explore the function of the increased miR-96 in hypoxic environment, we inhibited the up-regulation of miR-96 under hypoxia by transfection of different doses of miR-96I, and assessed miR-96 expression level (Fig 3B1), LC3-II accumulation (Fig 3B2) and cell proliferation (Fig 3B3). In addition, different doses of miR-96M were transfected and the above experiments were also performed. The results demonstrated that transfection of different doses (25-150nM) of miR-96M or miR-96I led to 0.01-4500 folds change (2^−ΔΔct^) of miR-96 expression (Fig. 3B1). When up-regulation of miR-96 under hypoxia was suppressed (about 2-3 fold), autophagy was also inhibited and LC 3-II decrease was more obvious when larger doses of inhibitors were transfected. In contrast, when miR-96M was transfected, the expression LC3-II was first increased and then decreased (Fig. 3B2). Cell viability test by CCK-8 kit demonstrated that large doses of miR-96M or miR-96I induced significant decrease of cell viability when autophagy was considerably inhibited. However, modest inhibition of miR-96 significantly increased cells viability in LNCaP cells (Fig. 3B3). Taken together, these results suggest that hypoxia-induced up-regulation of miR-96 might promote autophagy flux. However, further increase of miR-96 to a certain threshold might suppress autophagy and hence decrease cell viability.

### miR-96 regulates autophagy by directly targeting *MTOR* and *ATG7*

**Figure 4:**
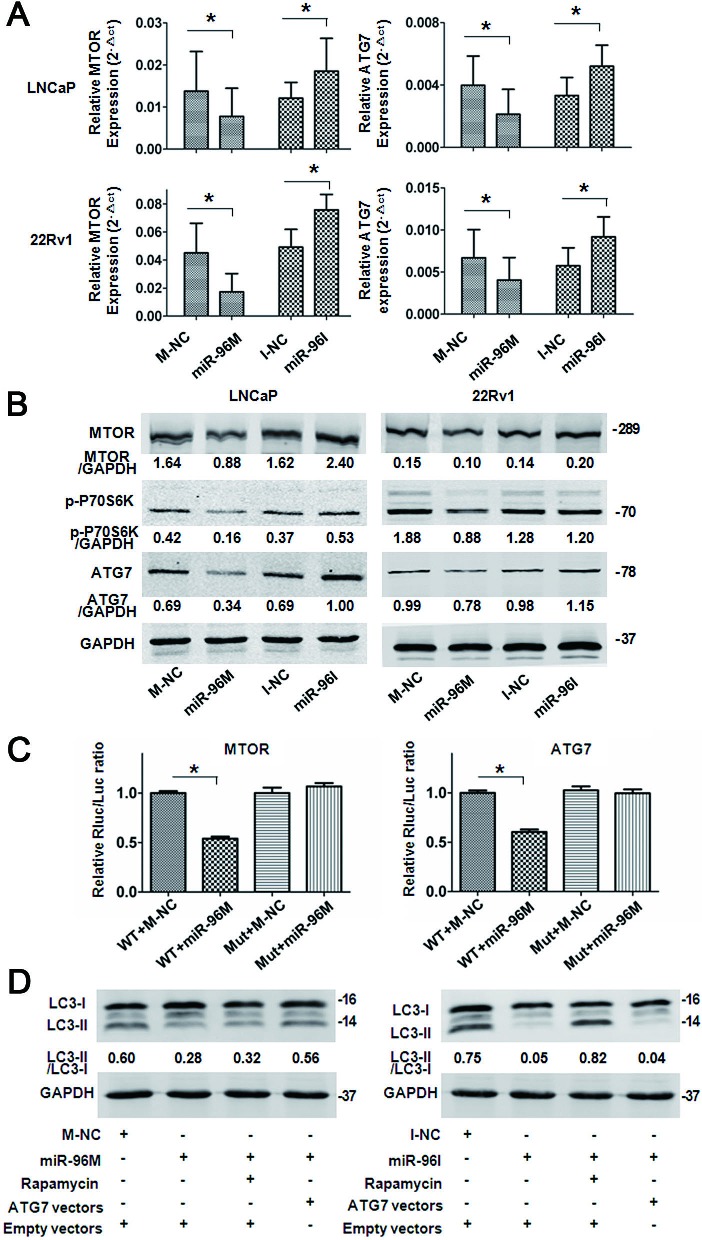
miR-96 regulated autophagy by targeting *MTOR* and *ATG7* A, LNCaP and 22Rv1 cells were transfected with 100nM miR-96M or miR-96I and incubated for 36 h. *MTOR* and *ATG7* mRNA levels were determined by qPCR (n=6). B, LNCaP and 22Rv1 cells were transfected with 100nM miR-96M or miR-96I and incubated for 36 h. *MTOR*, *p-P70S6K* and *ATG7* protein levels were checked by Western blots. C, Normalized luciferase activity in 293T cells co-transfected with the wild-type or mutant *MTOR* (*ATG7*) and negative control or miR-96M (n=3). D, Rescue experiments by over-expressing *MTOR* or *ATG7* in LNCaP cells. miR-96M (100nM), miR-96I (100nM), rapamycin (10nM), and CQ (50 M) was used in this experiment. M-NC: miRNAs mimics negative control; I-NC: miRNAs inhibitors negative control. *p < 0.05

To explore the mechanism by which miR-96 regulates autophagy, we searched “Targetscan” for the potential targets of miR-96 and identified 6 potential target genes including *ATG7*, *ATG16L1*, *ATG9A*, *MTOR*, *DEPTOR*, and *RICTOR*. We then evaluated the effects of miR-96 on those genes by transfecting miR-96M in LNCaP and 22Rv1 cells and checking the mRNA and protein levels of these genes with qPCR and Western blots. In both cell lines, we found that transfection of miR-96M resulted significant reduction of the mRNA levels of *ATG7* and *MTOR* (Fig. 4A). In contrast, transfection of miR-96I led to significant increase of the mRNA levels of *ATG7* and *MTOR* (Fig. 4A). Consistent with the alteration of mRNA levels, transfection of miR-96M and miR-96I resulted in significant reduction and increase, respectively, of the protein levels of ATG7 and MTOR (Fig. 4B). Phosphorylation of P70S6K, a direct downstream substrate of MTOR, was inhibited by over-expression of miR-96 (Fig. 4B), further supporting the inhibition of *MTOR* by miR-96. In addition, dual-luciferase reporter assays showed that over-expression of miR-96 significantly reduced the luciferase activity in cells transfected with plasmids containing wild-type but not mutant 3′UTR sequences of *ATG7* or *MTOR* (Fig. 4C).

We next asked whether miR-96 regulates autophagy through targeting *ATG7* or *MTOR*. Over-expression of miR-96 in LNCaP cells inhibited autophagy, whereas rapamycin did not reverse the effect of miR-96. However, co-transfection of miR-96M and plasmids with ATG7 cDNA rescued autophagy back to control levels (Fig. 4D left). In contrast, miR-96I also inhibited autophagy, which was reversed by rapamycin but not ectopic expression of *ATG7* (Fig. 4D right). These results together demonstrated that miR-96 regulates hypoxia-induced autophagy through targeting *ATG7* or *MTOR*.

**Figure 5:**
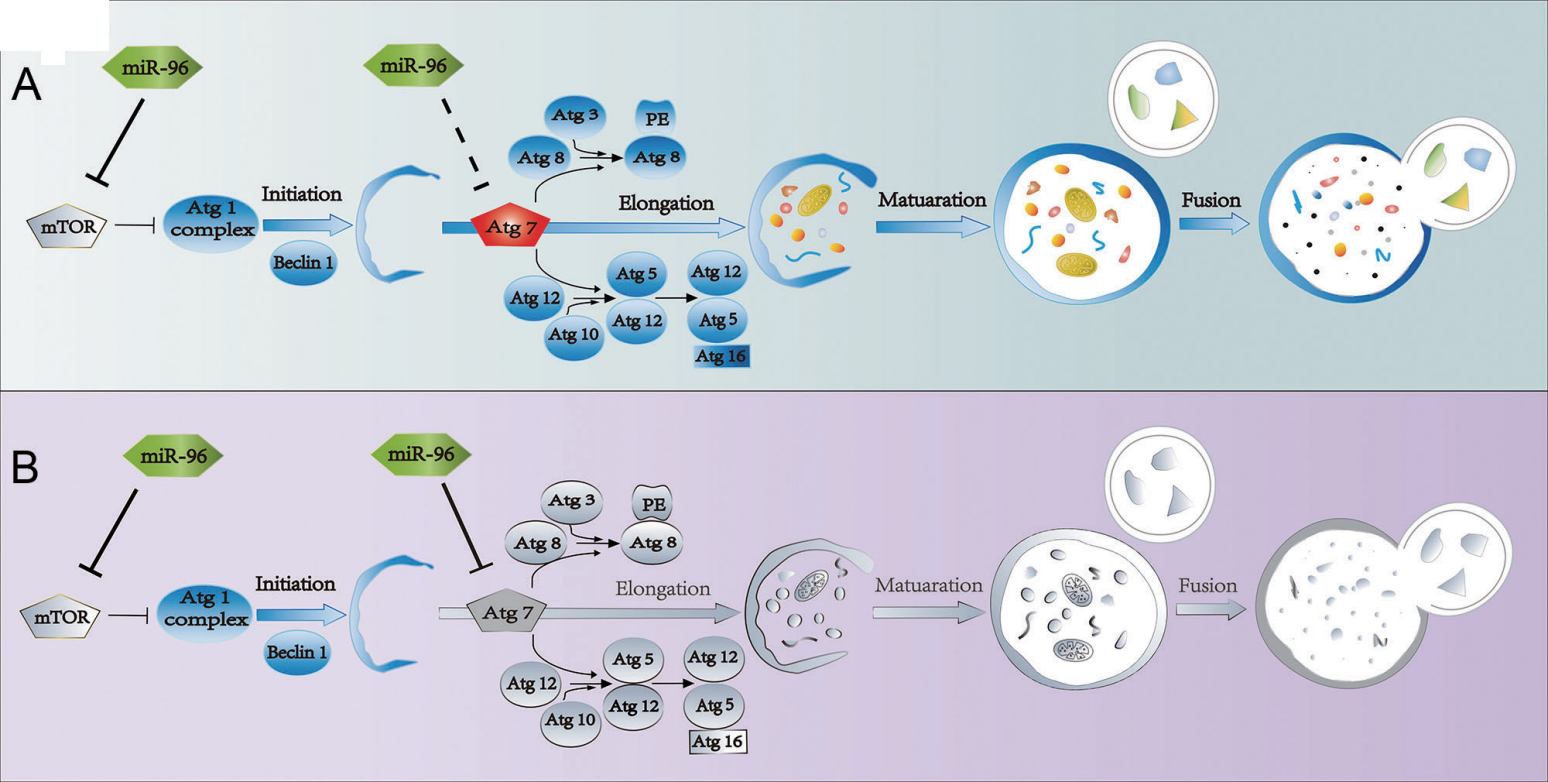
Schematic diagram demonstrating dose-dependent regulation of miR-96 on hypoxia induced-autophagy A, When miR-96 expression levels in LNCaP cells were 2^−Δct^ <8.6 or 2^−ΔΔct^ <1,000, while in 22Rv1 cells 2^−Δct^ <25 or 2^−ΔΔct^ <3,000, autophagy was promoted. B, When miR-96 expression levels in LNCaP cells were 2^−Δct^ >8.6 or 2^−ΔΔct^ >1,000, while in 22Rv1 cells 2^−Δct^ >25 or 2^−ΔΔct^ >3,000, autophagy was inhibited. Colorful draws represent active state of the factors, and gray draws represent the inactivated state.

*MTOR* inhibits autophagy while *ATG7* is essential for the formation of autophagosome. Targeting both *MTOR* and *ATG7* predicts that miR-96 can both inhibit and promote autophagy, indicating that miR-96 acts as both suppressor and promoter of autophagy. How does miR-96 modulate autophagy in such a contradictory way? Figure 3B1 and figure 3B2 demonstrated that, in LNCaP cells, when miR-96 level increased to the relative fold range 2^−ΔΔct^ <1,000, or the relative miR-96 expression level 2^−Δct^ < 8.6, autophagy was promoted. Similarly, in 22Rv1 cells, when miR-96 level increased to the relative fold range 2^−ΔΔct^ < 3,000, or the relative miR-96 expression level 2^−Δct^ < 25, autophagy was enhanced. However when miR-96 level was further increased, autophagy was suppressed. These results suggest the following model of the function of miR-96 in the regulation of autophagy: when miR-96 level increases at the first stage, the effect of inhibition on *MTOR* is more significant than that on *ATG7*, the outcome is the promotion of autophagy (Fig. 5A). However, when miR-96 continues to increase and exceeds the threshold, the inhibition on *ATG7* becomes the main event. Although a large dose of miR-96 might suppress *MTOR* expression and hence promote the initiation of autophagosome, the downstream autophagosome expansion is likely attenuated via inhibition of ATG7 by a large dose of miR-96. This may cause the final outcome of autophagy inhibition (Fig. 5B). Therefore, miR-96 regulates autophagy in a dose dependent manner in prostate cancer cells.

### Apoptosis and cell cycle shift contributes to the changes of cell viability

**Figure 6:**
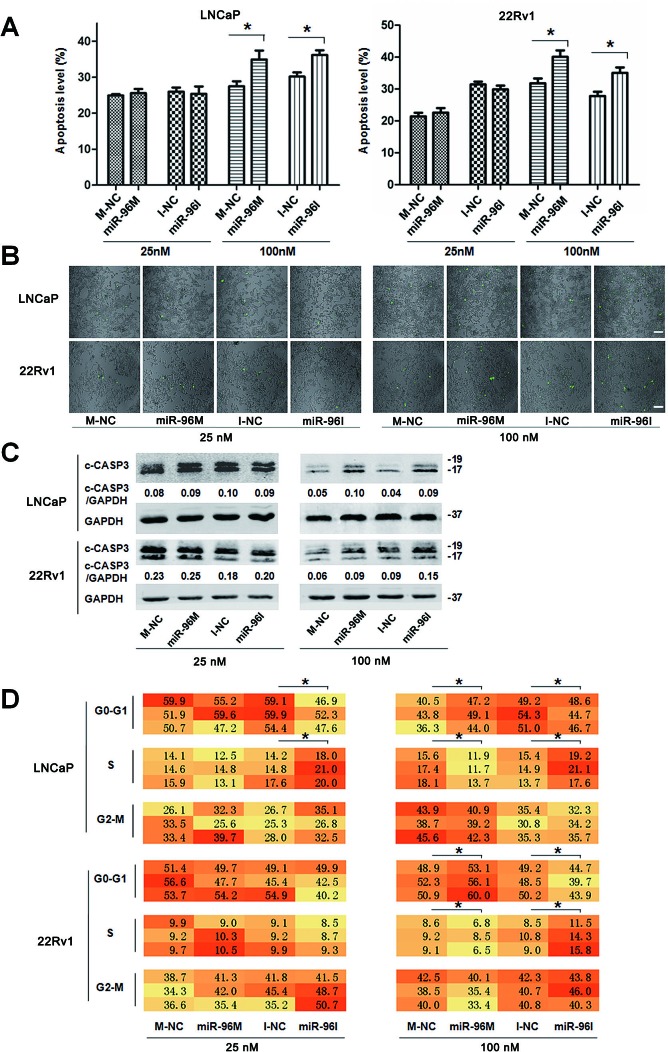
Apoptosis level and cell cycle shifted by miR-96 in prostate cancer cells subjected to hypoxia A, LNCaP and 22Rv1 cells were transfected with miR-96M or miR-96I at the indicated concentrations and incubated under hypoxia for 36 h. Apoptosis was examined by flow cytometer (n=3). B, LNCaP and 22Rv1 cells were transfected with miR-96M or miR-96I at the indicated concentrations and incubated under hypoxia for 36 h. CASP3 activity in adherent prostate cancer cells was tested. Green dots represented the cells in which CASP3 was activated. (Scale bars: 100 μm). C, Cells were treated as A, cleaved CASP3 was determined by western blots. D, Cells were treated as A, cell cycle distribution was determined by high-content screening. M-NC: miRNAs mimics negative control; I-NC: miRNAs inhibitors negative control. *p < 0.05

Autophagy may reduce apoptosis to help tumor cells to survive hypoxic stress [29]. In consistence, blockage of autophagy induces apoptosis under metabolic stresses such as hypoxia [10]. In addition, deregulation of autophagy or *MTOR* pathway leads to alterations of cell cycle distribution [32, 33]. We next assessed the apoptosis level and cell cycle distribution of LNCaP and 22Rv1 cells subjected to hypoxia for 36 h. Transfection of small doses (25nM) of miR-96M or miR-96I didn't change the rate of apoptotic cells (Fig. 6A), the activity of CASP3 (Fig. 6B), and the level of cleaved CASP3 (Fig. 6C). However, large doses of miR-96M or miR-96I (100nM) led to significant enhancement of apoptosis (Fig. 6A-C).

Hypoxia can induce cell cycle arrest [34]. To test if miR-96 affects cell cycle progression in hypoxia, we performed high-content screening systems. We found that miR-96I significantly restored cell proliferation in LNCaP cells subjected to 36 h hypoxia, regardless of transfection doses (Fig. 6D). In contrast, large dose of miR-96M induced a more significant cell cycle arrest in both LNCaP and 22Rv1 cells (Fig. 6D). These data suggest that the increase of cell viability by low dose of miR-96I (Fig. 3B3) may result from the restored cell proliferation by miR-96 inhibition, while large dose of miR-96I might decrease cell viability by enhancing apoptosis (Fig. 3B3). However, large dose of miR-96M may cause a significant decrease of cell viability by inducing both cell apoptosis and cell cycle arrest (Fig. 3B3).

### miR-96 expression level correlates with clinical parameters in prostate cancer tissues

**Figure 7:**
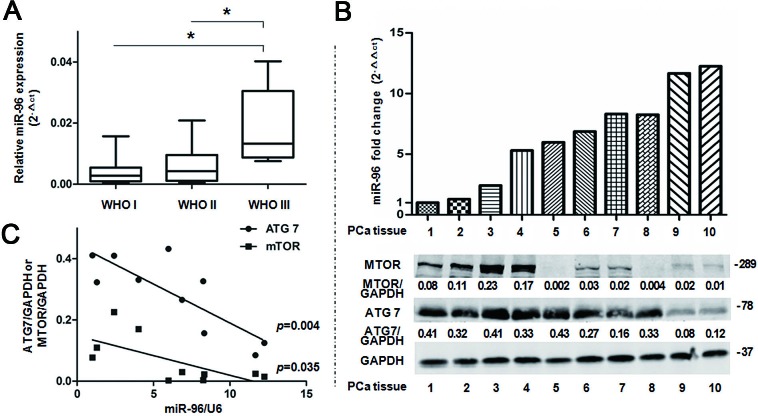
miR-96 expression level in prostate cancer tissues is correlated with clinical parameters A, miR-96 expression in the 20 prostate cancer tissues was determined by TaqMan qPCR analysis and grouped according to WHO I (n=8), II (n=7) and III (n=5) grade. Boxes represent the lower and the upper quartiles with medians; whiskers illustrate the 10 to 90 percentiles of the samples. B, Normalized miR-96 expression in the 10 prostate cancer (PCa) tissues was aligned with *MTOR* and *ATG7* protein levels determined by immunoblots. C, Quantitative analysis of the correlations between miR-96 expression and *ATG7* (r=0.82) or *MTOR* (r=0.67) expression in the above 10 prostate cancer tissues. *p < 0.05

To evaluate the role of miR-96 in prostate cancer, we detected miR-96 expression level and analyzed the relationship between miR-96 and patients' clinical parameters in 20 prostate cancer tissues. The results showed that miR-96 level increased with WHO grade with the mean miR-96 expression (2^−Δct^) of 0.004, 0.007, and 0.018 in WHO I, II and III grade, respectively (*p*<0.05) (Fig. 7A).

Further, we found that the maximum relative expression level (2^−Δct^) of miR-96 in the 20 prostate cancer tissues was 0.03 and the maximum fold change (2^−ΔΔct^) was 83, which indicate that miR-96 might promote autophagy (Fig. 3B) and serve as an oncogene in prostate cancer. In addition, great differences were found in the miR-96 expression level between prostate cancer tissues and the cells transfected with large dose of miR-96M.

To further investigate the relationship between miR-96 and *ATG7* or *MTOR*, we detected miR-96 expression level, ATG7 and MTOR protein levels in 10 prostate cancer tissues (Fig. 7B). We found that the miR-96 level was reversely correlated with the protein levels of ATG7 and MTOR (*p* < 0.05) (Fig. 7C), which indicate that miR-96 regulates *ATG7* and *MTOR* in prostate cancer tissues.

## DISCUSSION

In this study, we found that miR-96 regulated autophagy in prostate cancer cells in a dose-dependent manner through directly targeting two critical autophagy-related factors, *MTOR* and *ATG7*. Both *in vitro* and *in vivo* results demonstrated that regulation of autophagy by miR-96 affected prostate cancer cell proliferation and tumor growth. To the best of our knowledge, miR-96 is the first reported miRNA, which has a dual regulation on autophagy, providing a novel regulation mode of autophagy by miRNAs.

Both *MTOR* and *ATG7* are essential regulators in autophagy pathways. *MTOR* is an upstream factor and involves in the phosphorylation of ATG1/ULK1 and ATG13. *MTOR* not only modulates autophagy, but also controls numerous other biological processes such as cell proliferation, survival and metabolism. ATG7 is a downstream factor and participates in the formation of LC3-PE and ATG12-ATG5 conjugate. In this study, we found that miR-96 regulated autophagy through modulation of both *MTOR* and *ATG7*. It seems that when miR-96 increases in response to hypoxia, the effects of targeting *MTOR* play a more important role. However when miR-96 further increases and exceeds a certain threshold, the effects of targeting *ATG7* prevail.

miR-96 is a widely recognized oncogenic miRNA and was found to inhibit apoptosis [24, 35]. Our findings indicate that when miR-96 expression is elevated in response to hypoxic stress, it enhances autophagy and maintain cell survival by targeting *MTOR*. Tumor cells' response to hypoxia is mediated partly through the suppression of *MTOR* activity [36, 37]. Inhibition of *MTOR* in hypoxia may reduce cell metabolism and suppress cellular senescence [38], which is believed to be a self-protective mechanism to maintain energy homeostasis and cell survival. In addition, inhibition of *MTOR* enhances autophagy, which helps cells to eliminate defective organelles and molecules, thereby recycling nutrients for survival under hypoxia. Hypoxia inhibits the *MTOR* pathway by *REDD1* and the *TSC1*/*TSC2* tumor suppressor complex [37, 39, 40]. In this study, we found that miR-96 was increased under hypoxia, accompanied with decrease of both mRNA and protein levels of *MTOR*. Our data indicated that miR-96 is a new factor connecting hypoxia and *MTOR*. The hypoxic tumor microenvironment is a hallmark of most solid tumors. Hypoxia might increase the expression of miR-96, which in turn inhibits *MTOR* subsequently leading to decreased cell proliferation and nutrition consumption while enhanced cell survival. Although the hypoxia~miR-96~*MTOR* pathway need to be further investigated in a large-scale clinic analysis, our data suggest that the hypoxia-induced up-regulation of miR-96 is one of the mechanisms by which hypoxia suppresses *MTOR* signaling. In conclusion, the up-regulation of miR-96 in response to hypoxic stress in prostate cancer cells inhibited *MTOR*, enhanced autophagy, and maintained cell survival.

miR-96 is over-expressed in prostate cancer cells [23, 41] and was found to promote prostate cancer cell proliferation by suppressing *FOXO1* [35] and *SLC39A1* [23]. However, recently it was reported that over-expression of miR-96 could shift its oncogenic role towards inhibition of tumor growth [20]. But the underlying mechanism is largely unknown. In this study, we found when miR-96 further increased and exceeded a certain threshold, it shifted from its autophagy-promoting to autophagy-inhibiting function by predominantly targeting an essential autophagy-related gene *ATG7*. Although high level of miR-96 may inhibit *MTOR* and thereby promote the initiation of autophagosome, it will prevent the expansion of autophagosome, which depends on *ATG7*. However, we found great differences in the miR-96 expression levels between prostate cancer tissues and the cells transfected with large dose of miR-96M, suggesting that the inhibition on autophagy by miR-96 is probably an off target effect, which indicated *ATG7* might not be a target gene of miR-96 in prostate cancer tissues. However, we can't ascertain with confidence that targeting *ATG7* is an off-target effect, because miR-96 level was inversely correlated with the protein levels of *ATG7* (Fig. 7B, 7C) in prostate cancer tissues, which indicated miR-96 might target *ATG7* in prostate cancer tissues. Future studies are warranted to further elucidate the off-target effects of miR-96M [42] in a greater number of prostate cancer tissues.

It is not clear why inhibition of *ATG7* is not observed when the expression of miR-96 is below the threshold. Firstly, it is possible that the effects of miR-96 on *ATG7* are impaired by other potential factors or pathways when miR-96 expression level is below the threshold. Secondly, the dose-dependent regulation of autophagy by miR-96 might be due to the different binding affinity between miR-96 and *MTOR* or *ATG7*. It is possible the binding affinity between miR-96 and *MTOR* is stronger than that between miR-96 and *ATG7*, which is supported by the finding that *MTOR* scored higher than *ATG7* according to both “Targetscan” and “miRDB”. Consistent with our findings, miR-375, known as a tumor suppressive gene, was reported to target *PDK1*, leading to the inhibition of *PDK1*/*AKT*/*MTOR* pathway [43] and thus promotion of autophagy [44]. However, it was recently demonstrated that over expression of miR-375 targets *ATG7* and suppresses autophagy independently of its regulation on *PDK1*/*AKT*/*MTOR* signaling [45].

In summary, we found a dose dependent modulation of autophagy by miR-96 through regulation of *MTOR* and *ATG7*. These facts indicate that regulation of autophagy is complicated and flexible. Our data suggest that the regulation of miR-96 is in a dynamic balance in hypoxia, inhibition of *MTOR* by up-regulation of miR-96 may promote autophagy. However, ectopic over-expression of miR-96 to exceed a certain threshold may disrupt the balance and suppress autophagy. Of note, this biphasic regulation of autophagy by miR-96 affected prostate cancer cell proliferation and tumor growth. However, further studies are required to understand the complex role of miR-96 in autophagy regulation in hypoxia.

## METHODS

### Ethics Statement

Investigation has been conducted in accordance with the ethical standards and according to the Declaration of Helsinki and according to national and international guidelines and has been approved by the local institutional review board.

### Cell Lines and Patient Samples

Prostate cancer cell lines LNCaP and 22Rv1 were purchased from the Cell Bank of the Chinese Academy of Sciences (Shanghai, China) and recently authenticated based on cross species checks, DNA authentication and quarantine. LAPC4 cells were obtained from University of California at Los Angeles (UCLA) and authenticated as described [46]. Cells were cultured in RPMI 1640 medium (11875, Gibco, MD, USA) supplemented with 10% fetal bovine serum (10099, Gibco) at 37 °C in an atmosphere of 5% CO2 and 95% air. If cells were cultured in hypoxia, the oxygen was maintained at 1%.

Prostate cancer tissue samples were collected from 20 patients who underwent radical prostatectomy between 2011 and 2012 in Shanghai Renji Hospital (Shanghai, China) and stored in the biobank [47]. All cases were diagnosed using adjacent histopathological slice and graded according to the WHO standard. Informed consent has been obtained for all the patients.

### Chemical Reagents, miRNA Mimics, miRNA Inhibitors, Agomir, Antagomir, Plasmid, and Transfection

Chloroquine (CQ), 4′,6-Diamidino-2-phenylindole (DAPI) and rapamycin were obtained from Sigma-Aldrich, MO, USA (C6628, D9564, 37094). miRNA mimics, inhibitors, agomir, and antagomir were purchased from RiboBio, Guangzhou, China (miR10000095, miR20000095, miR40000095, miR30000095). ATG7 plasmid was purchased from GeneChem Technologies, Shanghai, China (POSE142067150). GFP-LC3 plasmid was constructed by the Central Laboratory of Renji Hospital. Plasmids were validated by DNA sequencing in Beijing Genomics Institute. Transfection of nucleic acids was performed using Lipofectamine 2000 (11668, Life Technologies, CA, USA) according to the manufacturer's instructions. When the effects of different doses of miRNA transfection were compared, the same dose of Lipofectamine 2000 was used according to the largest dose.

### RNA Isolation, cDNA Synthesis and Quantitative Real-time PCR (qPCR)

Total RNA isolation, cDNA synthesis and qPCR from prostate cancer cell lines were performed as previously described [48]. The primers for qPCR were listed in Supplemental Information Table S1. Small RNAs were extracted by mirVana^TM^ miRNA Isolation Kit (AM1561, Life Technologies). Reverse transcription and qPCR were conducted with TaqMan MicroRNA RT kit (4366596, Life Technologies) and TaqMan Universal Mastermix (4440043, Life Technologies), respectively. miR-96 specific primers were purchased from Life Technologies (Assay no. 000186). U6 snRNA (Assay no. 001973) was used as the reference gene.

### Western Blot

Protein from cell lines was isolated with M-PER Mammalian Protein Extraction Reagent (78501, Thermo Scientific, MA, USA). Protein from prostate cancer tissue was extracted with Complete Lysis-M (13354520, Roche, Mannheim, Germany). Protein concentration was determined by the BCA method (23225, Beyotime, Shanghai, China). Proteins were separated using 6-12% SDS-PAGE and transferred onto nitrocellulose membrane (10401396, GE healthcare, OH, USA). The membranes were blocked by blocking buffer (927-40000, Odyssey, MA, USA) for 1 hour and then incubated in the primary antibodies at 4°C overnight. After washing with TBST, the membranes were incubated with secondary antibodies at room temperature for 1 hour. Protein bands were visualized by infrared imaging system (Odyssey) and quantified with Odyssey application software. Rabbit anti-LC3B, SQSTM1, ATG7, MTOR, p-P70S6k, and cleaved CASP3 antibodies were purchased from Cell Signaling Technology, MA, USA (2775, 5114, 8558, 2983, 9208, 9664). Anti-GAPDH antibody was purchased from Abcam, Cambridge, UK (9485).

### Dual-luciferase Reporter Assay

A 1095-bp fragment of wild-type (WT) *ATG7* 3′UTR (Table S2) containing two conserved binding sites of miR-96 (Position 1287-1293 and 1984-1990) or a mutant *ATG7* 3′UTR sequence was cloned into the pmiR-RB-REPORT™ (RiboBio). For *MTOR* gene, the whole 3′UTR sequence (954-bp) containing one conserved binding site (Position 918-924) of miR-96 and a mutant sequence were cloned as for *ATG7* (Table S2). Different reporter vectors (*p*-hRluc-3′UTR-WT or *p*-hRluc-3′UTR-Mut) were co-transfected with miRNA mimics or mimics negative control into 293T cells. Luciferase activity was determined by Dual-Luciferase Reporter Assay (E1910, Promega, WI, USA).

### Cell Viability, Apoptosis and Cell-cycle Analysis

Cell viability was assessed with Cell Counting Kit-8 (CCK-8; CK04, Dojindo, Kumamoto, Japan) on a spectrophotometer (BioTek, VT, USA). Apoptosis was examined using the Annexin V-FITC Apoptosis Detection Kit (V13241, Life Technologies) and analyzed by FACSCalibur flow cytometer (BD Biosciences, CA, USA). CASP3 activity in intact adherent cells was detected with NucView 488 caspase-3 substrate (30029, Biotium, CA, USA). The stained cells were viewed with confocal microscopy. Apoptosis was also assessed by immunoblotting of cleaved CASP3. Cell cycle was analyzed with high-content screening by staining cells with DAPI. Cell cycle distribution was classified by integrated intensity.

### Confocal Microscopy and High-content Screening

LC3B immunofluorescence was detected with confocal microscopy as previously described [49]. GFP-LC3 transfection was analyzed with MD ImageXpress® Micro XLS System (Molecular Devices, CA, USA).

### Animal Experiments

All animal experiments were performed in accordance with the NIH Guide for the Care and Use of Laboratory Animals. We performed all animal surgery under ketamine anesthesia, and took every effort to minimize animal suffering. Athymic nude mice (J:NU, male; 6 weeks old; 20-30g) were provided by Shanghai SLAC laboratory Animal Co., Ltd. The mice were randomly divided into four groups (Agomir negative control group, n = 5; agomiR-96 group, n = 5; antagomir negative control group, n = 10; antagomiR-96 group, n = 10). LNCaP cells (5×10^6^) in PBS were inoculated subcutaneously into the flanks of nude mice. Tumor size was measured as described previously [45]. Four nmol agomiR-96 or antagomiR-96 in 50 μl PBS was directly injected into each tumor twice a week. The injection began when tumor volume reached 150-200mm^3^. Mice were sacrificed after 2 weeks.

### Statistical Analysis

Data analyses were conducted with SPSS 16.0 (SPSS, IL, USA) and GraphPad-Prism5 (GraphPad, CA, USA). Data are presented as the mean ± standard deviation (SD) and analyzed by the Student's t test or ANOVA. Pearson's correlation was performed to determine the relationship between miR-96 and its target genes. Schematic diagram was plotted with Adobe Illustrator CS6 (Adobe, CA, USA). Heat map was generated using Microsoft Office Excel 2007 (Microsoft, WA, USA). All tests were 2-tailed, and p < 0.05 was considered statistically significant. Each experiment was performed in triplicate.

## SUPPLEMENTARY, TABLE AND FIGURE


