# Supplementary material for: Biphasic regulation of autophagy by miR-96 in prostate cancer cells under hypoxia
Source: Oncotarget. 2014 Aug 27;5(19):9169–82. doi: 10.18632/oncotarget.2396 (PMC4253426; doi:10.18632/oncotarget.2396)
Supplement: Supplementary file 1 [file oncotarget-05-9169-s001.pdf]

Biphasic regulation of autophagy by miR-96 in prostate cancer cells under hypoxia

Supplementary Material

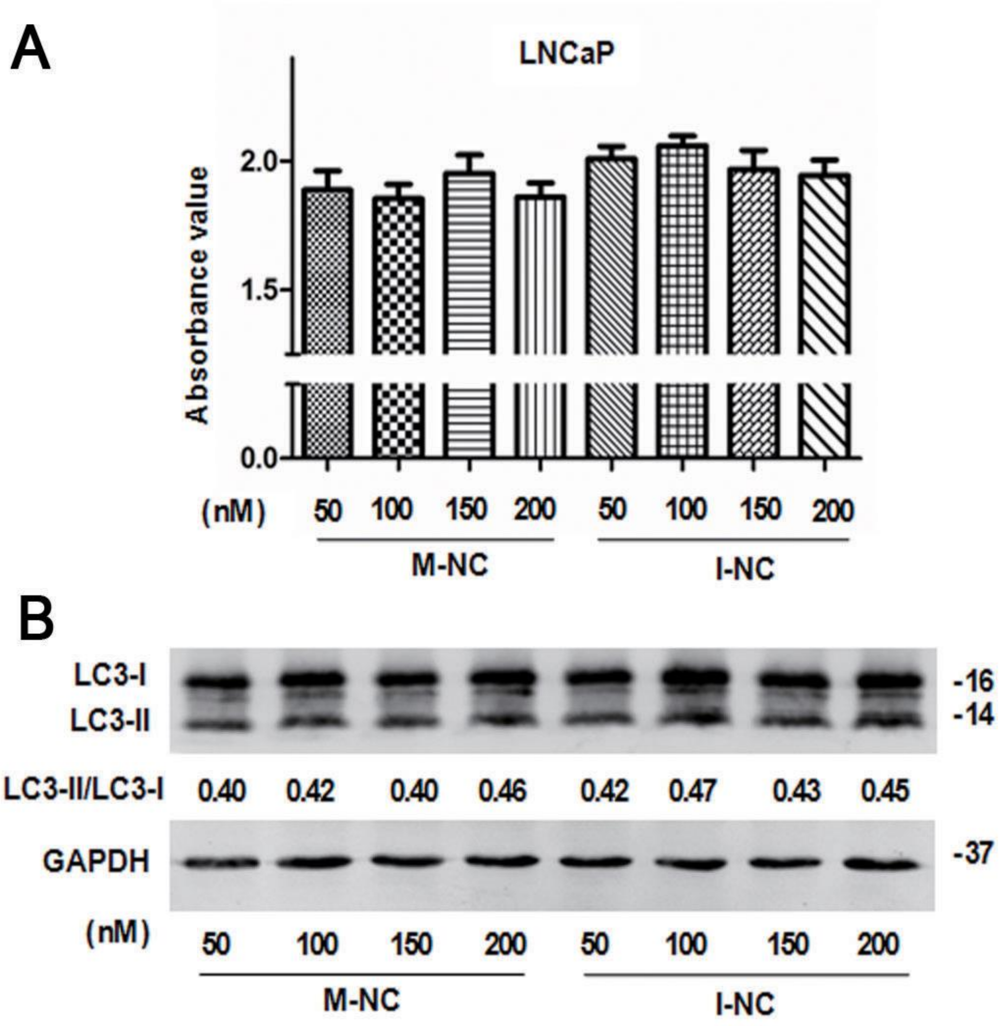

Figure S1: Increase in M-NC or I-NC concentration did not affect cell survival and autophagy. A, LNCaP cells were transfected with M-NC or I-NC with the concentrations as indicated. Cell viability was determined by CCK-8 after incubation in hypoxia for 36 h (n=6). B, LNCaP cells were transfected with M-NC or I-NC as indicated, and incubated under hypoxia with CQ (50 μM) for 36 h, LC3B expression was determined by Western blots. M-NC: miRNAs mimics negative control; I-NC: miRNAs inhibitors negative control.

Table S1: Primer sequences of *ATG7* and *MTOR*.

Gene Symbol	Forward primer (5'-3')	Reverse primer (5'-3')
ATG7	AGGAGATTCAACCAGAGACC	GCACAAGCCCAAGAGAGG
MTOR	GAACCTCAGGGCAAGATGCT	CTGGTTTCCTCATTCCGGCT

Table S2: *ATG7* and MTOR 3'UTR

***ATG7* 3'UTR:**

GATGGCCCCGCTGTGGGGCTGACTTCTCCCCGGCCGCCTGCTGAGGAGCTCTCCATC
GCCAGAGCAGGACTGCTGACCCCAGGCCTGGTGATTCTGGGCCCCTCCTCCATACCC
CGAGGTCTGGGATTCCCCCCTCTGCTGCCCAGGAGTGGCCAGTGTTCGGCGTTGCTC
GGGATTCAAGATACCACCAGTTCAGAGCTAAATAATAACCTTGGCCTTGGCCTTGCT
ATTGACCTGGGACTTGGTCCTCCATGCAGTTTTTATTTCTTGTCACAGTGACTGATAG
CCATCCCCCAGGATCCTTTCCCCTTGGCCCTGAGGGGGTGACCCAACACAGACCAAA
TGGGGAAATGAGCAACCAGCTCCTGCCCAGAGCCACTGCGGGAGGTGGCACCCTCA
TCCCCGGAATGTGCTGCCCACCGCACCGCAGGCTCCTCCTGTGGGGGCCCTGGGCAT
GGGTGAGGGTGGGACCCCGTGAGCGCACTGCACCCTGGCCCTGGTGGAGCGGGAGG
AGGAGGAGAGCCGAGCTGGGTACGAGACTAAAGGGCCCACATGACCCAGTGACGC
CAGATTTCCACCAAGGACTGAGTGAGCTGCTCAGACATGGCTTTCTGCCTCCCAGCC
TGTCCTCCACTGTGGGCATAGCATCTGTGCCTGCCTGCCTGCTTGAGGGAGAGGAGT
TTCTGCTGCTGCCTTGAGCTGGGGGGAAGAGCCCAGGGGCAGATCCTGGCAGCTGC
CTGGATGGGGCTCCTCCCTGCCCTTATGAGCAGGCCAGGCCCAGAAAGGCCGAGCC
TGGGCTGCCTTCCTGCCCCAGCCGAGGGAGGGGTCAGACGGCTCTACCATGGGTAA
CTCAGGCAAGAGCTGGTTTTCCTCTTTATTCTGGGTGTGTGCAGCTGTGAGGCCCCA
ACCCAGGAGAGGCCATGGCCTAGGTACCTGTGACCACCCTGCCCCCGTGTAGAGGG
CATCGTCTTTCCTGCTATTTTATTCTTTCAGCTTTTGTCTTAGGCCCAGAATCAAAGT
GAAAATTGAGTCGAGCTGACCCTTACAACAGTAGGATTTAGTAGGGTAGATTTCAA
ATGAGGCTTCGCTTCTCCCAAAGTAGCCAGTCCAAGTTCCAGTGGCTGTCGTTCAGC
TCATGGGAGCTTCATGGGGACACAGCCGGCACAGGTGCAGGGCCCGAGTCCGCCCA
CCCAGCCTGGCGCTGAAACTGCACACGTACACTATGTGGTTTAAGAGCACTTTATTA
TTGTTCTTAAGGCTACTTTTAAGTACAAAAAAAGATGGCCTGCCAAACCTTTTTTTTT
CTTCTTCCAGGAAAAACAGGCCACAGAGAATGGTATATTACAGATTTACACACATG
AAGAGAAGGTCAGAGCGCACTGCAGGCAGCGCGGCTCTGGGAAGAACTTCACGGA
GCCCCTTCTTAGAGCAGGGAGGGGGCTTTCTCAGTGAAATGTTTGGTTTTCTGCTGC
CTCCTCTGCCCCAGGCCCCCCTCCAGGGTACTGCCTATCCCAGATAGGTCAGTGCAC
CAGGGACCCGGCCGCCAGCACCGCCGACCCCTCCCAGAGTGACGCCCTTGTTCACTG
ACAAAGAGACCTGTCCCAGGAGTGTCCTCCACCGAGCCGGTCAGCTGTGGGTGGTTT

TCCTGTTACGACGCTCAGTAGCCTGTAGCAATAACAAACTCGTGGCTATGAATGCAG
ATGCAGTGTTCTCATAGAATAACTGTTCCTGCACTTTTACAGACAAATCTACGACAA
AAAAAAAGATCAACTTTTTTTTTCCGAACAACAAAAAAAATGAATGATTACAATAG
GAAAGGGAAAAATTAAATAGCTACATATCATTAACAAATTAATGTTCTTCAAAAAA
TACCTACAAATTTCTCTGTACATTCTTTACGCACAGCGTAACGATGGTCTCAAAATC
ACCCATATAGAAAAGTGTTCTCAACGATTTTTCCTACAGAAAATATAGGGGCCTGAA
TGCCAAAGCTTGGAAGCCCAGTACAGTGGGAGTGAAATGTGTGCGGGGCAAGGAGA
AGGGCTTTTCTTTCCTCCACTTTTCAAAGGCCTGCAGCCACTCTGTGACTACAAGAGC
CAGTCCTCCGACCTTTTCACCCAGTGCCAATTTCCAAAATTCAACAGCTAAAAACTG
TAAAACCGGGGGTCATACGGTGTGCAGAGTCCACAAAGCCTTGCAGGTGAGGTGAC
CACGCCCACGTCACCTGGTCAGGTGCCATCGTCGTGAGCCTCTGGTGGGCCAGGTGG
GACACAGCACACCCCAGGGGGAGGGGATAGAAACGCTCATTGACCAAAAAGGAGC
AGCTGTGACCTCCACAGCTGTGTCTGTCATGCTTGCTTCATCTAATTTCTAGTTAGTA
GCTATTAATATAGCAAATAATAAATGCAGTAATAACAGTATAAAGTCAGAGGAATG
TATACTGCCTTGGCCCCAGCGTACGAGGAAGCGTATAAAACACCATATCACAGATTG
TCTGTCAGTAATCTGCTGTTCAGCCAAGAGAGTTCAAAGGGAGCAGTTTCTGCATGT
AGGGAAGTTGGAAGACACAAACCCCACCTCCCCTGGGAGCTTGTAACAAAGCAGAC
AGGGATGCAAAAATAAATGATGTCAGCCTGCAGCCAAACTCCAGCATCCCACACCG
CAGCTGACCCACTGCTCATCGCGAGGGCCTGCCAGGAGCTGGCCTCCCGCACTACTT
GTGAGTAAAGTGAATATCAAATACCAATCTTAGAGTACAACTGTACCAGCAGTAAG
TATATCTAGGACTGTAACTGACAAAAATAAACTAATTCTGAAAAGAAAAAAAAAA

***MTOR* 3'UTR:**
CTGGAGGCCCAGATGTGCCCATCACGTTTTTTCTGAGGCTTTTGTACTTTAGTAAATG
CTTCCACTAAACTGAAACCATGGTGAGAAAGTTTGACTTTGTTAAATATTTTGAAAT
GTAAATGAAAAGAACTACTGTATATTAAAAGTTGGTTTGAACCAACTTTCTAGCTGC
TGTTGAAGAATATATTGTCAGAAACACAAGGCTTGATTTGGTTCCCAGGACAGTGAA
ACATAGTAATACCACGTAAATCAAGCCATTCATTTTGGGGAACAGAAGATCCATAA
CTTTAGAAATACGGGTTTTGACTTAACTCACAAGAGAACTCATCATAAGTACTTGCT
GATGGAAGAATGACCTAGTTGCTCCTCTCAACATGGGTACAGCAAACTCAGCACAG
CCAAGAAGCCTCAGGTCGTGGAGAACATGGATTAGGATCCTAGACTGTAAAGACAC
AGAAGATGCTGACCTCACCCCTGCCACCTATCCCAAGACCTCACTGGTCTGTGGACA
GCAGCAGAAATGTTTGCAAGATAGGCCAAAATGAGTACAAAAGGTCTGTCTTCCAT
CAGACCCAGTGATGCTGCGACTCACACGCTTCAATTCAAGACCTGACCGCTAGTAGG
GAGGTTTATTCAGATCGCTGGCAGCCTCGGCTGAGCAGATGCACAGAGGGGATCAC
TGTGCAGTGGGACCACCCTCACTGGCCTTCTGCAGCAGGGTTCTGGGATGTTTTCAG
TGGTCAAAATACTCTGTTTAGAGCAAGGGCTCAGAAAACAGAAATACTGTCATGGA
GGTGCTGAACACAGGGAAGGTCTGGTACATATTGGAAATTATGAGCAGAACAAATA
CTCAACTAAATGCACAAAGTATAAAGTGTAGCCATGTCTAGACACCATGTTGTATCA
GAATAATTTTTGTGCCAATAAATGACATCAGAATTTTAAACATATGTA

Red words: Cloned sequences

Yellow background: Conserved binding sites of miR-96
